# Miniaturized electromagnetic tracking enables efficient ultrasound-navigated needle insertions

**DOI:** 10.1038/s41598-024-64530-6

**Published:** 2024-06-19

**Authors:** Alexander Seitel, Daniel Groener, Matthias Eisenmann, Laura Aguilera Saiz, Bünyamin Pekdemir, Patmaa Sridharan, Cam Tu Nguyen, Sebastian Häfele, Carolin Feldmann, Brittaney Everitt, Christian Happel, Eva Herrmann, Amir Sabet, Frank Grünwald, Alfred Michael Franz, Lena Maier-Hein

**Affiliations:** 1 Division of Intelligent Medical Systems, German Cancer Research Center (DKFZ), 69120 Heidelberg, Germany; 2 Department of Nuclear Medicine, Clinic for Radiology and Nuclear Medicine, University Hospital, Goethe University Frankfurt, 60596 Frankfurt, Germany; 3 Department of Medicine, Institute for Biostatistics, Goethe University Frankfurt, 60596 Frankfurt, Germany; 4 Institute for Computer Science, Ulm University of Applied Sciences, 89075 Ulm, Germany; 5 National Center for Tumor Diseases (NCT), a partnership between DKFZ and Heidelberg University Hospital, 69120 Heidelberg, Germany; 6 Faculty of Mathematics and Computer Science, Heidelberg University, 69120 Heidelberg, Germany; 7 Medical Faculty, Heidelberg University, 69120 Heidelberg, Germany; 8 Helmholtz Information and Data Science School for Health, Karlsruhe/Heidelberg, Germany

**Keywords:** Biomedical engineering, Translational research, Computational science

## Abstract

Ultrasound (US) has gained popularity as a guidance modality for percutaneous needle insertions because it is widely available and non-ionizing. However, coordinating scanning and needle insertion still requires significant experience. Current assistance solutions utilize optical or electromagnetic tracking (EMT) technology directly integrated into the US device or probe. This results in specialized devices or introduces additional hardware, limiting the ergonomics of both the scanning and insertion process. We developed the first ultrasound (US) navigation solution designed to be used as a non-permanent accessory for existing US devices while maintaining the ergonomics during the scanning process. A miniaturized EMT source is reversibly attached to the US probe, temporarily creating a combined modality that provides real-time anatomical imaging and instrument tracking at the same time. Studies performed with 11 clinical operators show that the proposed navigation solution can guide needle insertions with a targeting accuracy of about 5 mm, which is comparable to existing approaches and unaffected by repeated attachment and detachment of the miniaturized tracking solution. The assistance proved particularly helpful for non-expert users and needle insertions performed outside of the US plane. The small size and reversible attachability of the proposed navigation solution promises streamlined integration into the clinical workflow and widespread access to US navigated punctures.

## Introduction

Insertion of needle-shaped instruments into the human body is a key procedure in the diagnosis and treatment of many diseases. One such approach is fine-needle aspiration to investigate suspicious lesions or for locally ablative treatment of malignant or benign tumors. During the frequently performed biopsy of thyroid nodules, for example, a thin needle is inserted into the relevant area of the thyroid gland and cells or fluid are aspirated by means of a syringe. In this type of interventions, precise placement of the needle is essential to avoid injury of surrounding structures, such as in this case the jugular vein or the trachea^1^.

Hence, such interventions are often performed under conventional US or computed tomography (CT) assistance. For needle guidance in thyroid punctures, US has emerged as the primary modality^2^ as it allows for real-time imaging of the anatomy, is widely available at a relatively low cost, and does neither expose the patient nor the physician to ionizing radiation. However, both US imaging and insertion of needle-shaped instruments are known to be expert tasks and require training^3^ not always available to physicians who only occasionally perform such procedures. Existing assistance solutions aim to augment the live US image with information on the needle location by means of optical^4–7^, (electro-) magnetic^8–10^, or image-based^11^ tracking. Regardless of the clinical application, localization (tracking) of the needle in the coordinate frame of the US image is essential to such guidance solutions. Approaches utilizing optical tracking for needle localization, such as the solution proposed by Sindram et al.^4^, rely on a configuration of special optical markers attached to the US probe and the needle, which are localized by means of a stereo camera system. While optical tracking allows for highly accurate localization of the attached optical markers, these approaches have several disadvantages. Usability and handling can often be restricted due to the bulky marker setup affecting both probe and needle. Additionally, the optical nature of the tracking requires a line of sight between the stereo cameras and the marker setup to ensure reliable localization of the instrument. Solutions based on EMT aim to alleviate these issues as they are not restricted by the line-of-sight requirement, and electromagnetic sensors attached to the US probe and needle typically have a small footprint. Several guidance solutions based on EMT have been proposed including the pioneering work of Krücker et al.^12^, and among others first approaches to guide ablation of thyroid nodules^13,14^. As EMT relies on measuring electrical currents induced in a sensor by an EMT source for localization^15^, it is susceptible to EMT field distortions, for example, those introduced by ferromagnetic objects in the tracking space. Thus, careful preparation of the area of intervention and setup of the tracking hardware is required that, if omitted, can result in unreliable localization if sources of distortion are not accounted for or if the tracking source is placed too far away from the sensors to be tracked. Image-based approaches^11^ have, to our knowledge, not been utilized in such guidance systems due to the susceptibility to tracking errors when the needle tip is not visible in the image. These inaccuracies are particularly challenging to avoid, given that even slight deflections from the optimal in-plane insertion can result in significant errors^16^.

**Figure 1:**
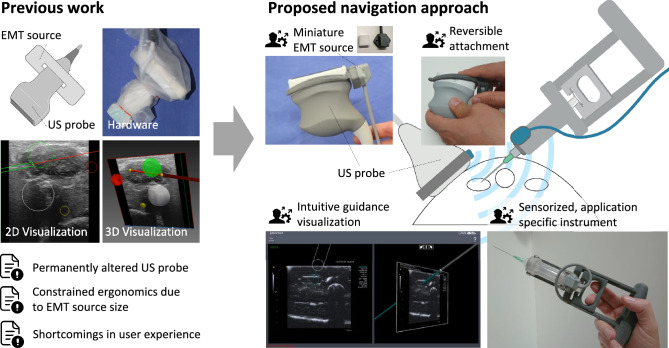
On-demand ultrasound (US) needle navigation enabled by miniaturized electromagnetic tracking (EMT). Left: Previous approaches result in permanently altered US probes and often constrained ergonomics due to the size of the EMT source. Right: In our proposed approach, a miniaturized EMT source (2.29 $\times$ 2.82 $\times$ 1.52 cm), marginally bigger than a sugar cube, is reversibly attached to the US probe in a known spatial relation using a specifically designed mounting bracket. The temporarily created combined modality of EMT and US imaging then allows for intuitive guidance visualization of the needle. The schematic illustration of the ultrasound probe, the biopsy needle and the phantom in this figure was created with Adobe Illustrator (Version 28.1, https://www.adobe.com/products/illustrator.html).

Most existing commercial US guidance systems such as LOGIQ E9 (GE Healthcare, Milwaukee, WI, USA), Virtual Navigator (Esaote Group, Genova, Italy) or PercuNav (Philips Healthcare, Hamburg, Germany, previously Traxtal Inc., Toronto, Canada) utilize EMT technology and can optionally be equipped with a device-specific needle guidance component. However, clinical adoption of such systems has been rather slow. Reasons may include additional complexity due to the setup of the hardware components, increased susceptibility to errors, and less ergonomic handling. To alleviate these issues, approaches have been proposed in which tracking hardware is permanently attached^5,6^ or integrated into^8,9^ US probes. However, when using an optical camera^5,6^, the instrument must always be located in a certain direction in relation to the US probe, while the localization range of a magnetized needle^8,9^ is limited. More recently, a new approach has been introduced that enables needle tip tracking for guidance in regional anesthesia based on measuring the US signal via a piezoelectric sensor embedded directly in the needle tip (Xperius^®^ Onvision^®^, B.Braun SE, Melsungen, Germany). First studies could show that this approach can improve peripheral nerve block procedures^17^. As only the tip of the needle is located, flexibility of needle and probe alignment can be restricted. Furthermore, with these concepts, tracking of structures other than the instrument is either difficult—requiring a second marker to be placed in the field of view of the camera^5^—or even impossible where only one magnetic object can be localized at the same time^8^.

Our earlier work saw us combining an EMT source with an US probe to be able to localize a number of miniaturized EMT sensors with a high degree of accuracy^18–20^. In initial studies, this concept showed promise for the targeting of liver lesions^20^ and thyroid nodules^19^ but was still limited by the dimensions of the setup and the necessity of permanently attaching the EMT source to the probe; affecting both the US scanning and needle insertion ergonomics.

Guided by user-centric design principles, we designed the first US navigation solution to be used as a non-permanent accessory for existing US probes, aiming to address both performance issues and workflow bottlenecks for a more seamless integration into clinical practice. Our main contributions are threefold: (1) We pioneer the use of a miniaturized EMT source for US-navigated needle insertions, addressing the ergonomic challenges originating from size constraints of earlier systems. The small footprint of the EMT source minimizes interference of the tracking hardware with the ultrasound scanning or needle insertion procedure. (2) We propose a concept for reversibly attaching this miniaturized EMT source to a US probe via a custom fit mounting bracket. This approach avoids the need for permanent attachment of tracking hardware and the necessity of specialized US probes, allowing existing US devices to be retrofitted with navigation functionality. The predefined setup at the ultrasound probe minimizes errors, as the area of highest tracking accuracy close to the EMT source coincides with the area of intervention, and reduces user-dependent, error-prone hardware setup before the intervention. (3) We evaluate the system’s performance through comprehensive assessments involving 11 clinical operators. Specifically, we investigate the achievable tracking accuracy with the attached miniaturized EMT source, determine the mounting precision of the attachment relative to the US probe, and assess the system’s guidance capabilities during a typical, clinically relevant interventional task.

## Material and methods

Figure 1 shows the overall concept of the US navigation solution. A miniaturized tracking device is reversibly attached to an US probe, temporarily creating a combined EMT and US imaging modality providing real-time anatomical imaging and instrument tracking at the same time. The localization information of the instrument equipped with a specialized tracking sensor can then be fed into a dedicated user-friendly interface that provides intuitive guidance information alongside the conventional US data.

Key to the approach is the reversible attachment of a miniaturized EMT source to an US probe, enabling tracking and imaging at the same time while preserving the ergonomics of the scanning process and the intervention in general. The main methodological concepts of this work, namely the miniaturized tracking for on-demand US guidance, a reliable fusion of US and tracking data including a novel verification concept, and the intuitive navigation interface are detailed in the following.

### Miniaturized tracking for on-demand US guidance

The navigation solution is based on a miniature EMT device (Polhemus TX-1, Polhemus Inc., Colchester, VT, USA), used here for the first time in the context of US-navigated needle insertions. The small size of the tracking source (2.29 $\times$ 2.82 $\times$ 1.52 cm, marginally bigger than a sugar cube, see Fig. 1) allows for reversible attachment to the US probe, a concept that had previously been unfeasible.

This reversible attachment is enabled by a mounting bracket that holds the tracking source at a fixed position and can easily be mounted to and unmounted from the US probe (see Fig. 1) and is inspired by needle guides such as CIVCO Ultra Pro (CIVCO Medical Solutions, Coralville, Iowa, USA)). To ensure a fixed placement, existing features of the US probe are used as mechanical anchors creating a probe-specific snap-fit connector. To maintain conventional ergonomics and handling of the US probe, the mounting bracket was chosen to be as small and resemble the shape of the US probe as closely as possible. The final design was reached after several iterations involving feedback from both technical assessments and clinical operators. The design was created using a 3D computer-aided design (CAD) software (Autodesk Inventor, Autodesk, San Rafael, CA, USA) and used as the input for 3D printing of the prototypes with a HP 4200 Multi Jet Fusion 3D printer (Hewlett-Packard Inc., Palo Alto, CA, USA) utilizing PA 12 (Polyamid 12) as printing material.

**Figure 2:**
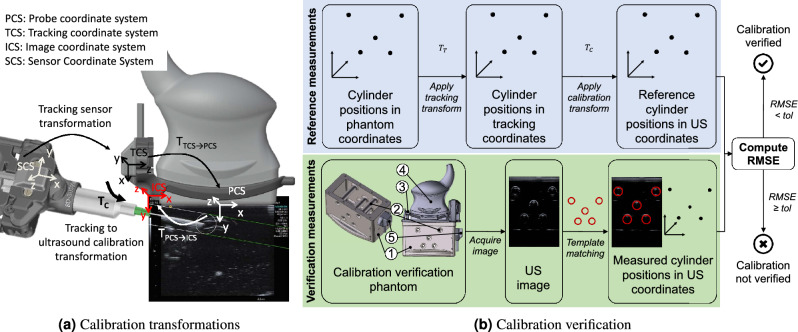
Tracking to ultrasound calibration. (**a**) Coordinate transformations involved in the calibration: $T_{PCS->ICS}$ between US probe and US image (fixed), $T_{TCS->PCS}$ between EMT source and US probe (fixed), final calibration transformation $T_C = T_{PCS->ICS}*T_{TCS->PCS}$ between US image and EMT source, the tracking sensor transformation (updated) can be used to visualize the needle relative to the live US image. (**b**) Ensuring quality through automatic calibration verification: (1) developed calibration verification device encloses (2) set of cylindrical rods, allows attachment of (3) combined modality of (4) ultrasound (US) probe and tracking add-on at a defined position to verify accuracy of configured calibration via (5) a reference tracking sensor attached to the housing of the phantom. Reference positions of the cylinders defined by design of the phantom. Calibration verified if the root-mean-square error (RMSE) between reference and measured points is smaller than a defined tolerance (tol = 2 mm). The 3D renderings in this figure were created with Autodesk Inventor 2018 (Version 22.0.11200.0000, https://www.autodesk.com/products/inventor).

### Reliable fusion of ultrasound and tracking data

The calibration transformation $T_C$ between the tracking coordinates and the ultrasound image coordinates, as shown in Fig. 2a, is determined given the knowledge about the image streamed from the US device, the US probe geometry and the geometry of the EMT source and its attachment. The streamed US image is first cropped to the image information important for the guidance visualization. The transformation between the US probe and the actual image coordinate system ($T_{PCS->ICS}$) is determined using these cropping parameters. The transformation between the EMT source and the US probe ($T_{TCS->PCS}$) is determined by the known geometries of the US probe, the EMT source and the mounting bracket. The design and the high printing accuracy (mechanical print resolution of 1200 dpi and a print layer height as low as 0.08 mm) allowed precise manufacturing of the mounting bracket. The final calibration transformation can then be calculated as $T_{C} = T_{PCS->ICS}*T_{TCS->PCS}$ based only on the US image parameters and mechanical hardware design instead of relying on manual calibration procedures^21,22^ such as N-Wire / Z-Wire approaches where an unambiguous wire pattern is used to determine the pose of the US probe from the image, wall-based approaches that employ a phantom in which the pose of the probe is determined by imaging a plane of known orientation, or point-based methods that calculate the probe transformation from a point structure attached to a phantom or a stylus that is imaged repeatedly.

The manual and reversible nature of the tracking solution’s attachment to the US probe, however, also entails the risk of possible inaccuracies. Mitigation of this risk is crucial for ensuring the safe usage in navigated US interventions. An appropriate quality control measure thus must allow for a quick and intuitive calibration accuracy check before each intervention and (1) provide features that can be accurately identified in both US and EMT coordinate space, (2) allow for placement of the US probe with attached EMT source at a known spatial relationship relative to the features, and (3) maintain the US image quality and the functionality of the EMT system. We thus introduce a calibration verification concept that consists of a *verification device* and an automatic *verification algorithm* for image-based calibration verification as shown in Fig. 2b.

The *calibration verification device* consists of a cuboid-shaped, watertight enclosure filled with US coupling gel that houses a set of five hollow cylinders. A connection mechanism holds the US probe with the attached tracking add-on in a defined position such that the US image vertically intersects the cylinders of the phantom. A tracking sensor attached at the side of the phantom establishes a reference measurement for the calibration verification algorithm.

To perform the *calibration verification*, the US probe with the attached tracking add-on is placed on the calibration verification device and both US images and tracking data of the phantom are continuously acquired. As reference measurements, the known positions of the phantom cylinders with respect to the tracking sensor are first transformed into tracking space by applying the currently acquired tracking transformation ($T_T$), and then transformed into US space by applying the calibration transformation to be verified ($T_C$). As verification measurements, the positions of the phantom cylinders are automatically localized in the US image via an iterative template matching process (OpenCV matchTemplate(), https://opencv.org, accessed on 03/15/2024) and compared to the reference measurements by computing the root-mean-square error (RMSE) between the two resulting point sets. If the RMSE is smaller than a set tolerance, the calibration is seen as valid and the calibration transformation determined from the mechanical design can be used for guidance. A failed verification indicates inaccuracies in the attachment of the bracket and the EMT source which need to be resolved before guidance is possible. The tolerance can be set according to the specific application need; here, it is chosen as 2 mm, approximately twice the expected error of the tracking system.

### User-centric ultrasound navigation interface

A central component is the user interface displaying the guidance information to the physician during the intervention. Based on the experience gained with the initial research prototype^18,19^, the following key requirements were identified for a user interface providing optimal navigation support while seamlessly integrating into the clinical workflow: (1) *Display of US image:* As an add-on to existing US devices, the user interface is required to display the US image as provided by the US system in real time and without perceivable latency. As navigation information is provided in a spatial context, visualization of the US image should be provided in both a 2D and 3D representation. (2) *Display of guidance information:* The information where the guided instrument is located in relation to the anatomical information shown in the US image must be displayed as overlaid guidance information. In particular, the prospective needle path and needle pose shall be visualized intuitively. (3) *Avoidance of user interaction during the intervention:* As the intervention requires the physician’s full attention to operating the US probe and needle, the need for user interaction during the intervention must be minimized.

**Figure 3:**
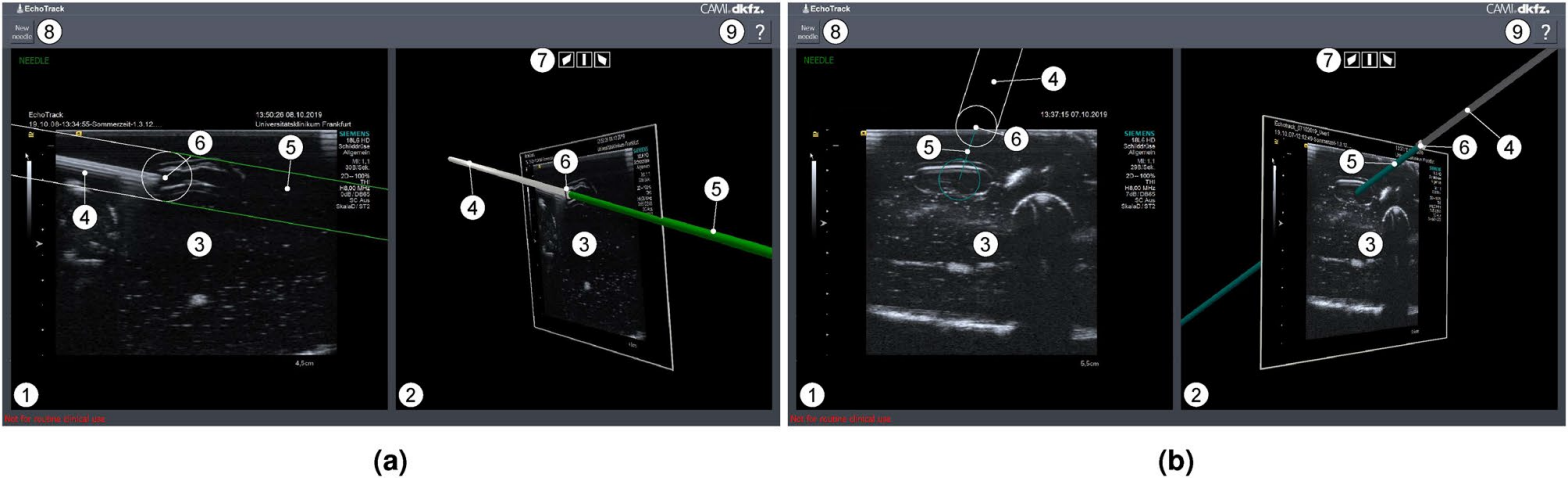
Graphical user interface of the guidance solution. Different visualizations are provided depending whether the needle is inserted (**a**) “in-plane” or (**b**) “out-of-plane”. A (1) two-dimensional and (2) three-dimensional visualization of the (3) ultrasound (US) image allows both for a conventional display of the US information and a display in the spatial context of the needle insertion process. The (4) needle and the (5) prospective path are depicted as a rendered surface model in the 3D scene and as a projection onto the US plane. The current position of the (6) needle tip is visualized between the needle and the prospective path. A quick setup of the (7) 3D scene allows to choose between predefined scene orientations (plane tilt left and right as well as view from the side). (8) Choosing a new needle triggers a calibration verification. Usage information can be obtained via a (9) help function.

The graphical user interface designed to fulfill these requirements is shown in Fig. 3. An example video is provided in the supplementary information (Supplementary Video V1). Its implementation based on the Medical Imaging Interaction Toolkit (MITK, www.mitk.org, accessed on 03/14/2024) followed a quality-centric development process including *test-driven development*, *continuous integration*, *code reviews* and final *acceptance testing* to mitigate errors introduced by the software itself. The live US image is both provided as a 2D and 3D visualization and augmented with a representation of the needle and an according visualization of the prospective path (2D: projection onto US plane, 3D: needle model in 3D scene) as well as a clear depiction of the currently targeted position on the US plane. Common presets for orienting the US plane in the 3D scene allow for a quick adjustment of the visualization.

#### In-plane guidance visualization

In ultrasound-guided needle insertions, in-plane insertion approaches are characterized by inserting the needle approximately parallel and very close to the ultrasound scanning plane^23^. During this approach, the physician tries to keep the needle in the scanning plane of the US probe, ensuring visibility of both the needle tip and shaft in the ultrasound image. This restricts the flexibility of probe orientation and possible needle approaches, but allows for image-based monitoring of the needle location with respect to the target and other anatomical structures, making it often the preferred approach for needle insertion. The in-plane guidance visualization supports this approach by providing information on the needle’s proximity to the US plane, its current location relative to the US plane (even if it is not yet be visible in the image), and the path it would follow if inserted from the current location and orientation (see Fig. 3a).

#### Out-of-plane guidance visualization

Needle insertion approaches are referred to as out-of-plane, if the needle is inserted in a direction that is not parallel and often perpendicular to the ultrasound scanning plane^23^. This enables a wide range of possible insertion paths and scan plane orientations, which is in particular beneficial for rather shallow targets in cases with restricted spatial access. Such cases render US visualization of the desired anatomy and performance of an in-plane insertion approach difficult. The proposed out-of-plane guidance visualization approach provides navigation support for such scenarios. It does so by visualizing the needle’s current location and pose and its prospective path in the combined 2D and 3D view and by providing additional cues helping in needle alignment in the 2D view (see Fig. 3b).

## Experiments and results

To assess whether the proposed miniaturized navigation solution can be used to assist US-guided percutaneous needle insertions, the influence of the design on the tracking accuracy, the achievable targeting accuracy and usability aspects were investigated for the exemplary use case of thyroid biopsies.

### Use case: thyroid biopsy

Thyroid biopsies are frequently performed as a diagnostic procedure for cytologic assessment of thyroid nodules. Typically, a thin needle is inserted into the tissue and a cellular specimen is extracted by aspiration via a syringe. Aspiration and handling during insertion are frequently supported by the use of a syringe holder^24^. To enable the proposed navigation solution for this use case, we developed a syringe holder capable of holding a tracking sensor and the syringe with the attached aspiration needle in a fixed spatial relationship (see Fig. 1).

**Figure 4:**
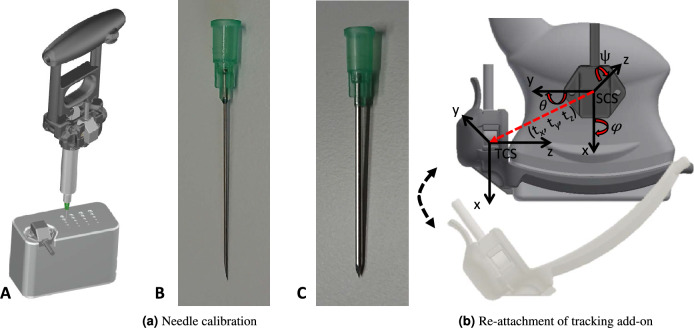
(**a**) Needle calibration device: (A) The needle attached to the syringe and syringe holder is advanced into the fitting insertion channel, for which the spatial relationship to an attached tracking sensor is known. The sought calibration transformation can then be calculated given the tracking data acquired by the electromagnetic tracking system. To assess the influence of needle bending on the needle calibration procedure, (B) a hypodermic needle, and (C) a modified prototype with a Kirschner wire to eliminate needle bending during the experiments was used. (**b**) Assessing the precision of the tracking add-on re-attachment. The tracking add-on is repeatedly attached to the ultrasound probe and the precision is determined for both the translational ($t_x$, $t_y$, $t_z$) and rotational ($\varphi$-, $\theta$-, and $\psi$- Euler Angles) components. The 3D renderings in this figure were created with Autodesk Inventor 2018 (Version 22.0.11200.0000, https://www.autodesk.com/products/inventor).

Localizing the needle tip based on the information provided by the tracking sensor was achieved through the introduction of a needle calibration device that allows for quick determination of the sought calibration transformation (see Fig. 4a). The needle calibration device holds 20 cylindrical channels of different diameters (from 1.0 to 3.5 mm) as well as an EMT sensor in a known and fixed spatial relationship. For calibration, the needle attached to the assembly of syringe and syringe holder was inserted into the fitting channel of the needle calibration device. Once fully inserted, 100 poses of the tracking sensor attached to the syringe holder as well as that attached to the needle calibration device were recorded and averaged to eliminate potential errors due to tracking jitter. Given the known transformation between the tracking sensors, the calibration transformation could then be calculated.

### Technical accuracy assessment

The assessment of the technical accuracy focused on experiments to determine (1) the tracking accuracy, (2) the ability to accurately re-attach the tracking add-on and (3) the use case-specific needle calibration accuracy.

#### Tracking accuracy

**Figure 5:**
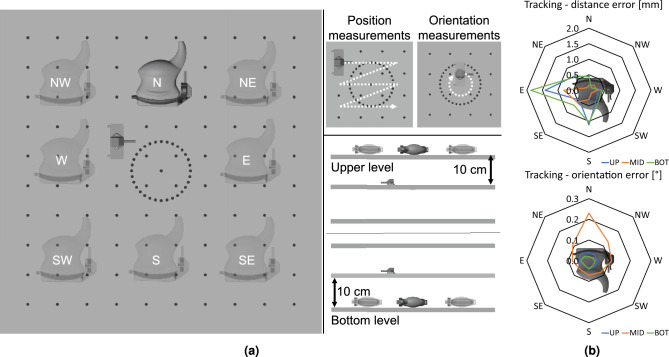
Tracking accuracy assessment using the Hummel protocol^25^. (**a**) Experimental setup. Left: The US probe with mounted tracking source is placed at eight positions on the Hummel board covering the tracking volume behind (SW, S, SE), in front of (NW, N, NE), and beside (W, E) the tracking source. Right, top: For each probe placement, tracking data is acquired from the tracking sensor at 12 positions of the 4 $\times$ 3 grid in the middle of the board and of 32 relative rotations between two consecutive sensor positions when rotated in the depicted circle. Right, bottom: The recording is performed with probe and sensor on the same level as well as shifted by 10 cm to the top and bottom, respectively. (**b**) Tracking accuracy of combined modality. Top: Distance error [mm] averaged over 17 measured distances of 5 cm per location on three height levels. Bottom: Orientation error [$^{\circ }$] averaged over 31 measured angles of 11.25$^{\circ }$ on three height levels. The 3D renderings in this figure were created with Autodesk Inventor 2018 (Version 22.0.11200.0000, https://www.autodesk.com/products/inventor).

An initial assessment of the tracking accuracy of the Polhemus TX-1 tracking source was presented in our previous publication^26^. It was found that the EMT is susceptible to interference from electrical devices and metallic objects, which is also known for other EMT systems^15^. However, one advantage of our concept shown in Fig. 1 is that the small EMT source is located directly at the relatively small working area and dynamic interference from objects other than the US probe is not to be expected. Here, we therefore focus on the system-specific accuracies. As in^26^, the standardized assessment phantom presented by Hummel et al.^25^ was applied to determine the tracking accuracy obtainable with the tracking source (Polhemus TX-1) attached to the US probe (Siemens 18L6HD) used for the ensuing evaluation. Positional measurements were taken by sampling tracking data from a positional grid of 4 $\times$ 3 = 12 positions, as illustrated in Fig. 5a.

On each of the 12 positions, *n* = 150 measurements were acquired. To determine precision, a jitter error at each position $p_i$ ($i\in \left\{ 1,\ldots ,12\right\}$) was defined as the RMSE of these measurements as in^26^. To evaluate the distance accuracy, the measured 9 horizontal (3 $\times$ 3) and 8 vertical (4 $\times$ 2) distances (total: 17) were compared to the reference distance of 5 cm. For this purpose, the 150 measurements were averaged for each position and the Euclidean distance between two averaged positions was computed. The deviation between the measured distance and reference of 5 cm was defined as the distance error $e_{distance}(d)$ for all 17 distances *d*. As a second measure for accuracy, the grid accuracy was determined by matching the 12 measurements to a set of reference positions with the optimal transformation in a least square sense^26^ and the fiducial registration error (FRE) of this transformation was used as grid matching error $e_{grid}$.

In addition, orientation measurements were taken by measuring 32 equidistant samples from the measurement circle on the board (Fig. 5a). For each orientation, 150 samples were recorded and averaged before further processing. The sensor direction on the sensor mount corresponds to ROT_1 from the publication by Hummel et al^25^. The orientational accuracy was then determined by comparing the angle between two successive measured orientations to a reference 11.25$^\circ$ (360$^\circ$ divided by 32 steps), leading to orientation errors $e_{orientation}(o)$ for each orientation $o\in \left\{ 1,\ldots ,32\right\}$.

As depicted in Fig. 5a, this tracking accuracy assessment was performed for different areas of the tracking volume, imitating sensor positions around the entire probe. For this purpose, the probe was placed at eight locations ${l\in \left\{ NW,N,NE,W,E,SW,S,SE\right\} }$ on the Hummel board, for three height levels $h\in \left\{ bot,mid,up\right\}$ each.

The tracking assessment summarized in Table 1 yielded a mean $e_{jitter}$ of 0.02 mm with a maximum of 0.14 mm over all 12 $\times$ 8 $\times$ 3 = 288 positions. While the jitter only varied marginally between the positions, locations, and height levels, the distance and orientation errors showed a slightly larger variation: As visualized in Fig. 5b, the largest $e_{distance}$ (up to 4.19 mm) and $e_{grid}$ (up to 2.73 mm) were found for locations E on all levels, when the probe is located between tracking source and sensor. Averaged over all locations on all levels (8 $\times$ 3 = 24), the mean $e_{distance}$ was 0.54 ± 0.44 mm (mean ± standard deviation) and the mean $e_{grid}$ was 1.15 ± 0.76 mm. The $e_{orientation}$ was found to be 0.07$^{\circ }$ averaged over all 8 $\times$ 3 $\times$ 31 = 744 relative distances with a maximum of 0.64$^{\circ }$.

**Table 1:** Results of tracking assessment for each location regarding jitter (mean RMS in mm), distance (mean in mm) and grid matching (in mm) as well as orientation errors (mean in $^{\circ }$).

Measurement level	Metric	NW	N	NE	E	SE	S	SW	W
Upper level (UP)	$e_{jitter}$	0.01	0.02	0.03	0.03	0.04	0.02	0.02	0.02
	$e_{distance}$	0.11	0.44	0.65	1.41	0.44	1.12	0.36	0.31
	$e_{grid}$	0.42	0.97	1.58	2.31	1.29	2.23	0.97	0.57
	$e_{orientation}$	0.04	0.04	0.05	0.06	0.03	0.04	0.04	0.04
Mid level (MID)	$e_{jitter}$	0.01	0.01	0.02	0.02	0.03	0.02	0.02	0.02
	$e_{distance}$	0.23	0.16	0.11	0.81	0.54	0.3	0.12	0.27
	$e_{grid}$	0.36	0.26	0.36	1.87	1.06	0.6	0.23	0.42
	$e_{orientation}$	0.13	0.23	0.11	0.08	0.06	0.07	0.08	0.11
Bottom level (BOT)	$e_{jitter}$	0.02	0.02	0.03	0.02	0.04	0.02	0.02	0.01
	$e_{distance}$	0.11	0.46	0.61	1.87	0.68	1.05	0.46	0.41
	$e_{grid}$	0.48	1.07	1.78	2.73	1.95	2.19	1.2	0.77
	$e_{orientation}$	0.02	0.02	0.03	0.04	0.03	0.04	0.03	0.03

#### Re-attachment of tracking add-on

Of central importance for the proposed navigation solution is that repeated placement of the tracking add-on does not negatively influence the overall accuracy of the whole system. The precision of the add-on attachment was determined by means of a temporarily attached additional reference sensor (Polhemus RX2) at the US probe body (see Fig. 4b). The add-on was repeatedly attached to and detached from the US probe (50 times) and the precision was determined both for the positional (x-, y-, z-axis) and the orientational ($\varphi$-, $\theta$-, and $\psi$- Euler Angles) components of the measured transformation provided by the tracking sensor. The positional precision was determined as 0.07 mm (X), 0.06 mm (Y), and 0.03 mm (Z), the orientational precision as 0.02$^{\circ }$ ($\varphi$), 0.03$^{\circ }$ ($\theta$), and 0.09$^{\circ }$ ($\psi$).

#### Needle calibration accuracy

To assess the accuracy of needle calibration, a pivoting test inspired by the calibration method described by Yaniv et al.^27^ was performed. Therefore, the needle tip of the used instrument, i.e., the trackable syringe holder with syringe and needle, was placed in a small cavity on a flat surface. While the tip was in a fixed position, the syringe handle was rotated approximately three times to collect 100 tracking samples of the tip. For a perfectly calibrated needle, the tip should always be in the same position. The needle calibration error was defined as the deviation of the tracked tip positions and measured as the RMSE of the position of the 100 tracked samples.

As bending of the hypodermic needle attached to the syringe is expected to increase the error, experiments were also performed with a modified prototype where the needle was replaced by a Kirschner wire to avoid bending, as shown in Fig. 4a. Nevertheless, even with the standard needle, efforts were made to avoid bending as much as possible, because deliberate bending could easily produce a large error. The procedure described above was performed for both needle types in three independent test runs.

The results of the calibration experiment with the Hyperdemic needle are errors of 1.10 mm, 1.03 mm and 0.86 mm (average 1.00 mm). When using the Kirschner wire without bending, the errors of 0.87 mm, 0.83 mm and 0.87 mm (average 0.86 mm) were slightly smaller.

### Targeting accuracy

**Figure 6:**
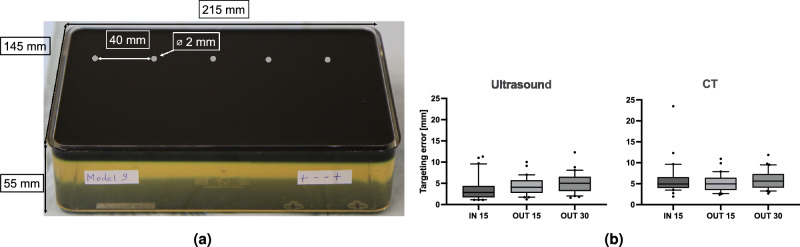
Targeting accuracy assessment. (**a**) Phantom used to assess targeting accuracy: five metal bead targets were placed in a container at 15 or 30 mm depth and a distance of 40 mm embedded in ballistic gelatin. (**b**) Targeting error for both out-of-plane insertions at 15 and 30 mm depth (OUT 15, 30) and in-plane insertions at 15 mm depth (IN 15) measured both in the acquired ultrasound and computed tomography images.

To assess the targeting accuracy, gelatin models (Gelita, Eberbach, Germany) were prepared (Fig. 6). Five metal target beads (2 mm diameter, rolling-element bearing steel, G.A.P. Kugellager, Enger, Germany) were positioned in 15 and 30 mm depth to correspond to locations typically encountered in clinical scenarios applying US-guided fine-needle thyroid punctures. Three clinical operators of different levels of experience in fine-needle aspirations (< 50, 50–100, and > 100 interventions performed so far) aimed to insert a puncturing needle (21G, 40 mm B. Braun, Melsungen, Germany) as closely as possible to each target using EMT guidance. A dedicated protocol mandated replicate punctures (n = 10) using both out-of-plane and in-plane approaches for targets at 15 mm depth, and out-of-plane punctures for targets at 30 mm depth. A single puncture without repositioning was carried out for each target. Once the needle reached its closest position to the target along the chosen insertion path, it was detached from the syringe. The distance from the needle tip to the center of the targeted metal bead was measured in a post-interventional CT scan. To identify whether a needle was displaced whilst being transferred to the CT scanner, the distance was determined by US measurement (Siemens Acuson 1000, Erlangen, Germany) prior to moving and once again by CT scanning (CT component of a Siemens Biograph 6 PET/CT scanner, Erlangen, Germany). MITK was used for management and measurement of the acquired CT images.

A total of 90 punctures were carried out by the 3 operators (performing 10 punctures in the three above-mentioned approaches each). Results are summarized in Fig. 6b. In-plane punctures were performed with targets placed at a depth of 15 mm (IN 15) and resulted in a mean targeting error of 3.8 ± 3.0 mm and 6.0 ± 4.0 mm for US and CT measurements, respectively. The marked discrepancy between US and CT measurements is most likely attributable to a small number of outliers due to unintentional movement of the needle during transportation or syringe detachment. For out-of-plane punctures at 15 mm depth (OUT 15), US and CT measurements showed comparable results with a targeting error of 4.3 ± 2.2 mm and 5.2 ± 2.2 mm, respectively. At 30 mm (OUT 30), targeting errors of 5.1 ± 2.5 mm and 5.9 ± 2.4 mm were observed.

### Assessment of clinical use in a thyroid phantom

**Figure 7:**
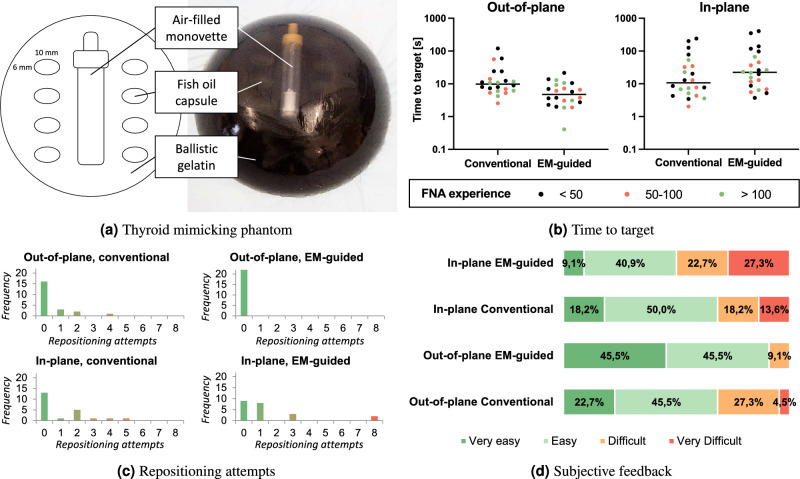
Evaluation for clinical use case. (**a**) Phantom for clinical usability assessment. Eight ellipsoidal fish oil capsules (10 $\times$ 6 mm) were embedded as targets in a semi-spherical mass of ballistic gelatin. The capsules were arranged on both sides (4/4) of a centrally placed monovette used to mimic tracheal sparing. Black color was added to the gelatin mass to prevent direct visibility of the targets. (**b**) Time from needle insertion to successful target aspiration for all punctures, out-of-plane and in-plane approaches. (**c**) Repositioning attempts and (**d**) feedback reported on a 4-point scale stratified by puncturing approach.

Clinical usability was examined in a pilot study with eleven clinical operators performing targeting experiments in a thyroid-like phantom. As depicted in Fig. 7a, the phantom was designed to mimic the puncture geometry of four right-sided and four left-sided thyroid lesions. Ballistic gelatin (Gelita, Eberbach, Germany) served as embedding mass for the target lesions (10 $\times$ 6 mm ellipsoidal gelatin-coated oil capsules, Schaebens, Frechen, Germany) placed at a depth of approximately 10 mm. An air-filled monovette (Sarstedt, Nümbrecht, Germany) was added to simulate tracheal sparing.

The puncture protocol for each participant included four right-sided and four left-sided punctures, incorporating both transisthmic in-plane and multi-angle out-of-plane approaches. Half of the punctures for each approach were assisted by the proposed EMT guidance solution, the other half conducted in the conventional way. Prior to performing the punctures mandated by the study protocol, all participants underwent a structured 20 minute training session to become familiar with the basic use of EMT guidance in a test phantom. A puncture was defined as successful if the operator was able to aspirate content from the targets. The number of repositioning attempts and time-to-target (TTT; time from needle insertion to target aspiration) were documented by two observers. Subjective feedback was given on a four-point-scale (ranging from very easy to very difficult). The eleven operators were categorized by their level of experience in thyroid fine-needle aspiration (FNA): 5 with less than 50 FNAs, 3 with 50 to 100 FNAs, 3 with more than 100 FNAs performed. A total of 88 data points was available for analysis (11 participants $\times$ 8 punctures). SPSS (Version 27.0.0.0, IBM, Chicago, IL) was used to perform a linear mixed effects analysis. As fixed effects, the use of EMT guidance, puncturing approach (out-of-plane/in-plane), and level of experience were incorporated. As random effects, participating individuals were added to the model. TTT was included as dependent variable of interest after log10-transformation. Two-way-interaction was examined for the use of EMT guidance and puncturing approach. A Wilcoxon signed rank test was used to compare subjective feedback with *p*-values <0.05 denominating statistical significance.

The results (Fig. 7b) show that a low FNA experience level was associated with significantly higher TTT values by a mean factor of 2 (*p* = 0.017) when compared to users with high experience levels. In contrast, the TTT for a moderate FNA experience level did not significantly differ from that of a high experience level (*p* = 0.897). For EMT-guided punctures, significantly higher TTT values were observed for in-plane compared to out-of-plane approaches by a mean factor of 5.7 (*p* < 0.001). In contrast, there was no significant difference between in-plane and out-of-plane puncturing time for controls (*p* = 0.379). The interaction of the chosen puncturing approach and use of EMT guidance was significant (*p* = 0.003). This interaction is also reflected by the observation that differences in puncturing time between controls and EMT-guided punctures were not significant for in-plane approaches (*p* = 0.116) whilst punctures were carried out in significantly shorter time with EMT guidance for out-of-plane approaches by a mean factor of 2.5 (*p* = 0.008). The use of EMT guidance for out-of-plane approaches also lowered the number of repositioning attempts (*p* = 0.026) and subjective feedback on a four-point-scale was improved (*p* = 0.019), as shown in Fig. 7c,d.

## Discussion

To our knowledge, we are the first to present a needle guidance solution that can be used as a non-permanent accessory for existing US systems. At the core of our approach lies a miniature EMT source that is reversibly mounted to conventional US probes, temporarily transforming a regular clinical US device into a US-based navigation system. In studies with 11 clinical operators, we could show that the navigation solution is especially helpful for needle insertions performed outside the US plane. Non-expert users in particular benefited from the provided guidance information. Targeting errors were found to be around 5 mm, which is comparable to existing navigation approaches. Compared to currently available solutions, our proposed guidance approach presents two key advantages:

1. *On-demand US needle navigation:* existing conventional US systems can be temporarily equipped with the proposed solution. Specialized US probes or permanently attached tracking hardware are not necessary.
2. *Compact and ergonomic design:* undisturbed US scanning and handling of the puncturing needle is enabled by the small size of the tracking attachment.

In the following paragraphs, we discuss the individual contributors to the overall performance which we determined in our comprehensive assessment of the navigation approach.

*Miniaturized tracking:* Tracking accuracies of the miniaturized EMT source attached to the US probe were determined in a controlled setting via the standardized protocol of Hummel et al.^25^ and found to be 0.54 mm and 0.06$^{\circ }$ on average. Compared to our previous setup using NDI (Northern Digital Inc., Waterloo, Canada) tracking technology attached to an US probe, similar results for the position measurements (0.6 mm^18^) and even better results for the orientation measurements (1.7$^{\circ }$ (ROT_1)^19^) could be achieved. The measured jitter also remained inconspicuous with less than 0.14 mm. Previously published baseline measurements^26^ assessing the tracking accuracy of the standalone EMT source report accuracies of 0.6 mm and 0.1$^{\circ }$ on average, indicating that the reported accuracies in our setup are already in the achievable accuracy range of the used tracking hardware. It needs to be noted that the protocol of Hummel et al.^25^ has limitations. Its design only allows for sparse, manual sampling of the tracking space, which is in particular evident for assessing the rotational component of the tracking accuracy for which only recordings of a circle in the center of the tracking volume are considered. Cable routing during these 360$^\circ$ measurements can also lead to problems. We accounted for those limitations by performing measurements at several different positions in the tracking volume. Future work should nevertheless concentrate on precise automatic assessment methods that allow for dense sampling of the whole volume. Furthermore, as can be seen in Fig. 5b, tracking is to a certain extent affected by the US probe, resulting in lower accuracies on the side of the EMT source obstructed by the probe body. At this point, it is worth noting that similar interference can also be caused by metallic objects in the working area, e.g. up to approx. 0.2–0.3 mm deviation due to aluminum, bronze or stainless steel cylinders in a related setup with the Polhemus TX-1 source^26^. However, such objects are usually not to be expected in the small working area of the US probe and, in contrast to setups with larger tracking volumes, it is easier to remove them. Additionally, it needs to be mentioned that for different US probes distortion properties and thus achievable accuracies might differ. Due to the fixed spatial relation between EMT source and US probe, distortion compensation approaches can be a promising means to minimize the static portion of the introduced distortion. Improvements in tracking hardware might additionally help to overcome these issues. In summary, while tracking accuracy for the proposed approach can still be further improved, it is currently already at a level close to what is reachable with the available tracking hardware. Its contribution to the overall error can thus be seen at an expected level.

*Image and tracking data fusion:* Fusion of US imaging data and EMT data is central to the proposed navigation solution. The available 3D models of the US probe, the EMT source as well as the mounting device allowed for a direct computation of the tracking to US calibration transformation, thus avoiding errors introduced by often manually performed calibration procedures such as N-Wire/Z-Wire, wall-based, and point-based approaches^21,22^. Reported calibration accuracies achieved when applying the calibration transformations determined by such methods are in the range of 1–2 mm^22^. With our calibration verification approach, we were able to confirm that applying the calibration transformation directly determined from the mechanical design consistently results in calibration accuracies below the chosen tolerance of 2 mm. We could also show that the errors introduced by the reversible attachment of the EMT source to the US probe were marginal, attachment could be performed with a precision of less than 0.1 mm and 0.1$^{\circ }$. A comprehensive analysis of the calibration errors is challenging as highly accurate reference measurements are difficult to obtain. In most cases they rely on phantoms that are tracked by an additional sensor which itself warrants a calibration procedure which is subject to potential errors. Errors for our direct computation of the calibration transformation are most possibly caused by inaccuracies in the 3D model of the US probe, the EMT source and the developed mounting bracket. Small improvements might be possible through more accurate 3D modeling of the US probe which was currently achieved by manual reverse engineering. We believe, however, that, given the high construction accuracies, achievable improvements are marginal.

*Instrument localization:* The localization of the needle in tracking space is highly dependent on the application and the corresponding instrument used. For the use case of thyroid biopsies investigated in this study, tracking of the hypodermic needle was only possible via a sensor attached to the syringe holder as opposed to direct tracking of the needle tip. Bending of the fine aspiration needles used in this clinical scenario is likely to have substantially influenced the accuracy of the guidance overlay and with it the overall targeting accuracy. During calibration of the needle tip relative to the attached tracking sensor, we addressed this issue by using a reference sensor-based calibration method which allows for a more controlled calibration environment than widely used pivoting methods. Nevertheless, our needle calibration experiment (cf. Fig. 4a) showed that needle bending still influences the calibration accuracy. In general, as reported by related studies^2,28,29^, needle bending remains an issue and could also not be completely avoided in our case, which potentially led to higher targeting errors or an increased number of repositioning attempts based on the additional information provided by the US image. We thus assume that the most significant improvements in tracking accuracy of the miniaturized EMT-based navigation solution can be achieved by a more accurate and reliable localization of the needle. In particular direct tracking of the needle tip could mitigate this issue. In our previous work^18^, in which we applied the NDI Aurora (Northern Digital Inc., Waterloo, Canada) technology, we were able to achieve targeting errors around 3 mm when using a needle that allows direct tracking of its tip. The smallest sensor technology available for the EMT system used in this work (Polhemus Micro Sensor 1.8) does currently not allow for tip tracking of fine needles. Related EMT systems (e.g. NDI Aurora or 3D Guidance technology as used e.g. in CIVCO VirtuTRAX, CIVCO Medical Solutions, Coralville, Iowa, USA) do. However, these approaches do not yet provide the possibility for miniaturized tracking as proposed in this paper. Risk stratification systems for thyroid nodules advocate performing ultrasound-guided fine needle aspiration in suspicious nodules with diameters $\ge 10$ mm (in high risk nodules)^30^. Given this widely accepted minimal threshold for nodule biopsy, the achieved puncturing accuracy can be considered reasonably sufficient to yield intranodular specimens for further diagnostic workup in the examined clinical scenario.

*Targeting and usability:* The navigation solution proved particularly helpful for out-of-plane punctures where both the number of needle repositioning attempts as well as the intervention time could be reduced. This is also reflected in the participants’ feedback, with none reporting the guided out-of-plane approach to be “very difficult”. The in-plane approach, on the other hand, was experienced as at least “difficult” by half of the participants. These difficulties can be attributed to several factors. As opposed to the out-of-plane approach for which the intersection of the prospective path and the US plane results in a single, clear intersection point that needs to be aligned with the target visible in the US image, the in-plane approach warrants an alignment of the whole path with the US plane (see Fig. 3). This turned out to be inherently more difficult for the study participants, as already small deviations of the needle from the optimal in-plane trajectory led to a lack of needle visibility in the US image. Furthermore, the errors introduced by the system have likely amplified this issue. In some cases, the provided overlay guidance visualization indicated the needle to be aligned with the US plane, while it was still not visible in the US image. We believe that this challenging hand-eye coordination significantly contributed to the decreased performance for the in-plane approach. Needle bending as discussed in the previous paragraph could have further amplified this issue. Based on the feedback from the users, a refined visualization scheme should focus more on indicating the current area in which the tip is located and on guiding the operator to the in-plane pose, which can then be verified directly in the US image.

In summary, the main challenges that currently prevent the proposed navigation solution from achieving a higher targeting accuracy are related to a more accurate localization of the used puncturing instrument, in particular if flexible needles are involved. As this is highly dependent on the dedicated clinical application, further research and development should concentrate on methods for accurate needle tracking while still maintaining the increased ergonomics and usability of the proposed miniaturized EMT solution for US-navigated needle insertions.

## Conclusion

For the first time, US-navigated needle insertions are enabled by a reversibly attached miniature EMT source temporarily equipping conventional US devices with a navigation functionality essential for precise and accurate targeting. By this means, widespread access to US-navigated punctures becomes feasible resulting in reduced trauma by effectively avoiding injury to structures at risk, and increased diagnostic yield as biopsies can be performed more efficiently.

### Supplementary Information

Supplementary Legends.

Supplementary Video 1.

## Data Availability

The datasets generated during and/or analyzed during the current study are available from the corresponding author on reasonable request.
