# Supplementary material for: Miniaturized electromagnetic tracking enables efficient ultrasound-navigated needle insertions
Source: Sci Rep. 2024 Jun 19;14:14161. doi: 10.1038/s41598-024-64530-6 (PMC11187124; doi:10.1038/s41598-024-64530-6)
Supplement: Supplementary file 1 — Supplementary Legends. [file 41598_2024_64530_MOESM1_ESM.pdf]

Supplementary Material

Supplementary Video V1

User interface of the proposed ultrasound navigation solution. The enabled needle guidance is showcased for both the in-plane and the out-of-plane insertion approach. The schematic illustration of the ultrasound probe, the biopsy needle and the phantom in this video was created with Adobe Illustrator (Version 27.3, https://www.adobe.com/products/illustrator.html).
